# Experience and Insight Into Genetic Diagnosis of Infective Aortic Aneurysm

**DOI:** 10.1002/ccr3.71586

**Published:** 2025-11-30

**Authors:** Shinnosuke Fukushima, Hideharu Hagiya, Takumi Fujimori, Koji Iio

**Affiliations:** ^1^ Department of General Medicine Okayama University Graduate School of Medicine, Dentistry and Pharmaceutical Sciences Okayama Japan; ^2^ Department of Bacteriology Okayama University Graduate School of Medicine, Dentistry and Pharmaceutical Sciences Okayama Japan; ^3^ Department of Infectious Diseases Okayama University Hospital Okayama Japan; ^4^ Microbiology Division, Clinical Laboratory Okayama University Hospital Okayama Japan

**Keywords:** 16S rRNA, culture‐negative, *Enterobacter hormaechei*, infected aneurysm

## Abstract

Culture‐negative infected aneurysms possibly occur in various clinical situations, including prior antibiotic exposure. Accurate microbial identification is crucial for an optimal antimicrobial strategy. 16S ribosomal RNA gene sequence analysis would provide a useful tool for precise bacterial identification.

A 73‐year‐old man undergoing chemotherapy for metastatic duodenal cancer presented to another hospital 5 months prior with fever. A contrast‐enhanced computed tomography upon admission showed polycystic liver abscesses and an infected aortic aneurysm measuring 41 mm in maximum diameter (Figure 1A–C). Bacterial cultures of blood and drainage specimens from the liver abscesses identified *Streptococcus intermedius* . The liver abscesses were managed through a combination of antibiotics and percutaneous transhepatic drainage, while the infected aneurysm was treated conservatively. Following discharge from the previous hospital, he received oral cefpodoxime and underwent outpatient monitoring. Despite stable inflammatory markers, sequential imaging demonstrated progressive expansion of the infected aneurysm (Figure 1D–F), necessitating admission to our hospital for aortic graft repair surgery. The infected thoracoabdominal aneurysm was approached through a left thoracoabdominal incision. Endovascular repair was not selected because complete resection of infected tissue is essential to prevent recurrence. The culture of the excised aortic tissue specimen was negative; therefore, antibiotic susceptibility testing could not be performed. Pathological findings showed scattered necrotic material within the aortic wall, accompanied by infiltrations of lymphocytes and macrophages, suggesting a chronic inflammatory process and fibrotic changes. Postoperatively, the patient received parenteral cefotaxime, resulting in significant clinical improvement. The patient was eventually transferred to a rehabilitation facility for continued care and subsequently discharged after a total of 6 weeks of postoperative intravenous antibiotic therapy without recurrence (Figure 2).

**FIGURE 1:**
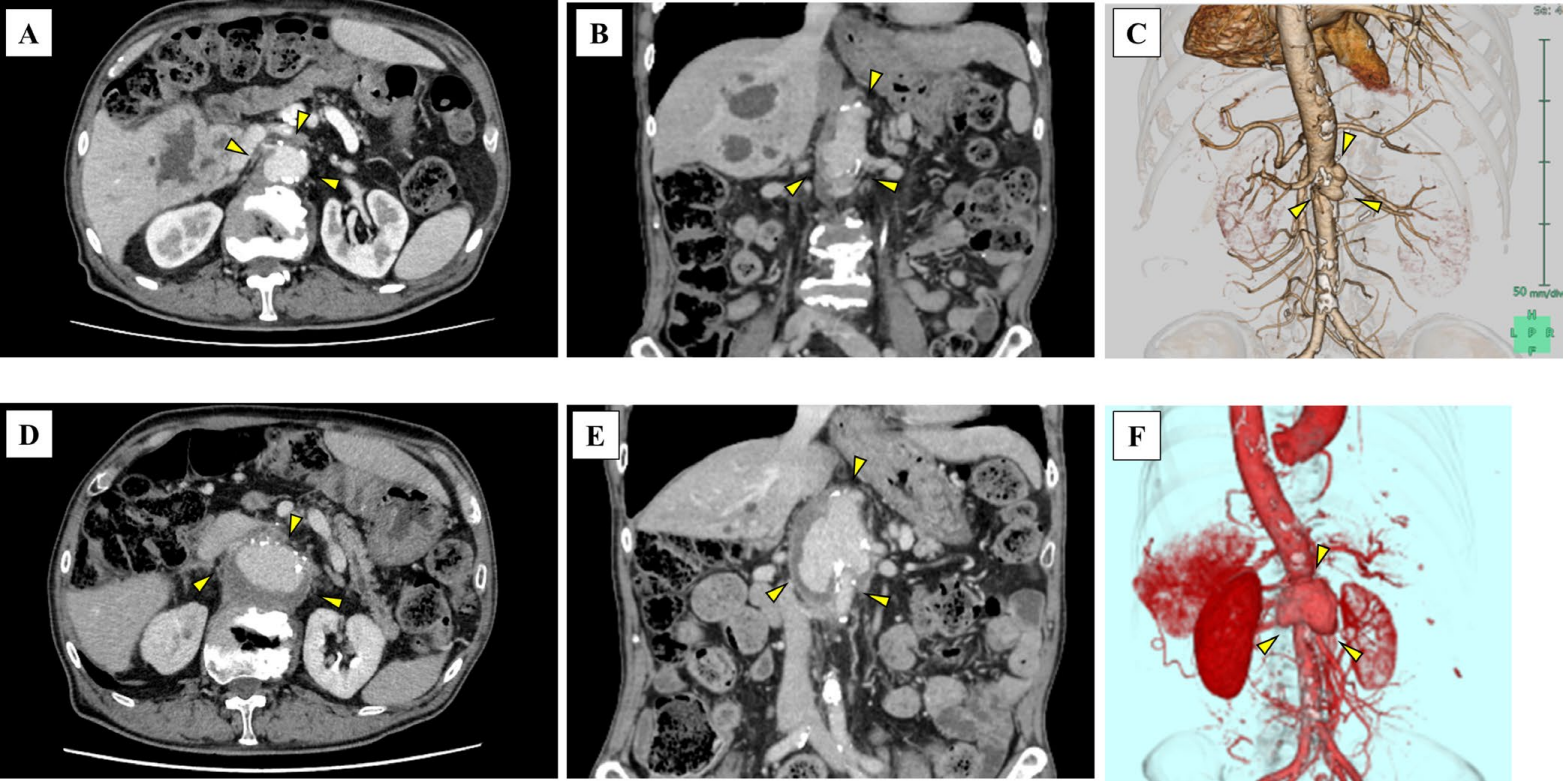
Contrast‐enhanced computed tomography (CT) showing a developing infected aortic aneurysm. (A–C) Contrast‐enhanced CT showing polycystic liver abscesses and an infected aortic aneurysm at 41 mm in maximum diameter (the arrowheads). (D–F) Contrast‐enhanced CT showing an infected aortic aneurysm at 60 mm in maximum diameter (the arrowheads).

**FIGURE 2:**
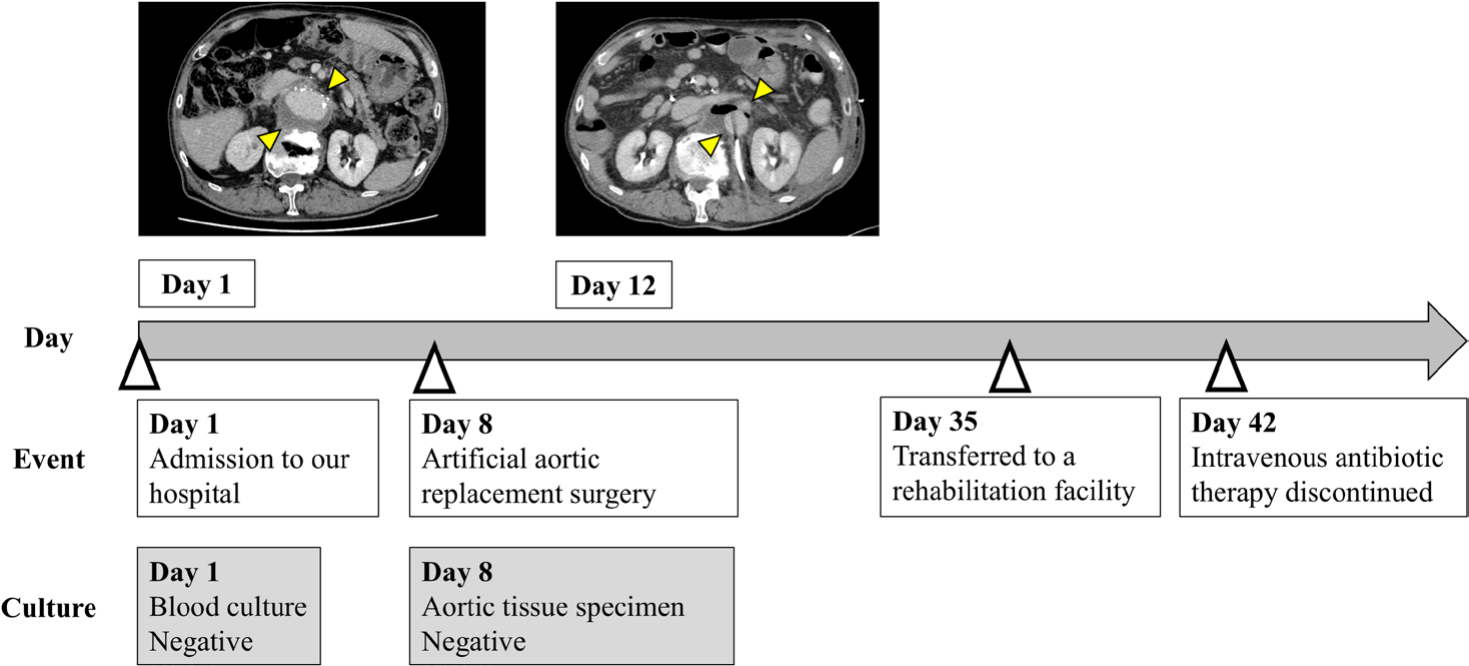
Clinical course of the case. Blood and aortic cultures were negative, and the postoperative clinical course was uneventful. After surgery, the patient underwent intravenous antibiotic treatment for 6 weeks.

Later, molecular identification of the bacterial pathogen was performed utilizing PCR‐based methodology. First, we extracted DNA from the resected aorta, using the DNeasy PowerSoil Pro Kit (QIAGEN), and then performed a two‐step PCR amplification protocol for 16S ribosomal RNA (rRNA) gene sequencing. The target gene was amplified with 8UA and 1485B primers (forward primer: 5′‐AGAGTTTGATCMTGGCTCAG‐3′; reverse primer: 5′‐TACGGTTACCTTGTTACGAC‐3′). The second PCR process was carried out with 341A and 519B primers (forward primer: 5′‐CTACGGGAGGCAGCAGTGGG‐3′, and reverse primer: 5′‐ATTACCGCGGCKGCTG‐3′). The sequence data of the PCR product was analyzed using the Basic Local Alignment Search Tool (BLAST). The result showed a 98.95% concordance with the reference strain of *Enterobacter hormaechei* (GenBank accession number: CP017186.1).

We described a clinical case wherein *E. hormaechei* was successfully identified as the causative bacterium through 16S rRNA gene sequencing in a tissue specimen from a culture‐negative aortic aneurysm. The predominant etiological agents in infective aortic aneurysms include *Staphylococcus aureus* and *Salmonella* species [1], whereas members of Enterobacterales are less commonly associated with the pathogenesis of this fatal disease. Antimicrobial‐resistant *E. hormaechei* isolates, including those producing carbapenemases, have been reported worldwide [2]. While accurate microbial identification is crucial for providing appropriate therapy, culture‐negative infected aneurysms occur at an unexpectedly high prevalence of 42% among documented cases [1]. Recent literature demonstrates the successful application of 16S rRNA gene sequence analysis in identifying causative pathogens in such cases [3]. Advancements in molecular diagnostic techniques facilitate the detection of pathogens that remain unidentifiable through conventional methodologies. Accurate identification of the etiological organism enables optimization of antimicrobial therapeutic strategies, particularly when managing infections caused by those potentially harboring extensive drug resistance profiles.

## Author Contributions

**Shinnosuke Fukushima:** writing – original draft. **Hideharu Hagiya:** conceptualization, writing – review and editing. **Takumi Fujimori:** investigation, writing – review and editing. **Koji Iio:** investigation, writing – original draft.

## Funding

The authors have nothing to report.

## Consent

Written informed consent was obtained from the patient for the publication.

## Conflicts of Interest

The authors declare no conflicts of interest.

## Data Availability

The datasets used during the current study are available from the corresponding author on reasonable request.
